# Computer-assisted preoperative planning of reduction of and osteosynthesis of scapular fracture: A case report

**DOI:** 10.1515/med-2021-0338

**Published:** 2021-10-25

**Authors:** Roman Madeja, Grzegorz Bajor, Ondrej Klima, Lubor Bialy, Jana Pometlova

**Affiliations:** Institute of Emergency Medicine, Faculty of Medicine, University of Ostrava, Ostrava 703 00, Czech Republic; Trauma Center, University Hospital Ostrava, 17. listopadu 1790/5, Ostrava 708 52, Czech Republic; Department of Human Anatomy, Faculty of Medical Science in Katowice, Medical University in Katowice, Katowice 40-752, Poland; IT4Innovations Centre of Excellence, Brno University of Technology, Brno 708 00, Czech Republic; Department of Medicine of Disasters, Faculty of Medicine, University of Ostrava, Ostrava 703 00, Czech Republic

**Keywords:** scapula, comminuted fracture, TraumaTech software, reconstruction, implant design

## Abstract

**Introduction:**

Reports on the use of computer-assisted trauma surgery of comminuted scapula fracture are still quite rare. In this article, we present a case of comminuted scapula fracture, the surgical reconstruction of which was pre-operatively planned using a complex software solution.

**Materials and methods:**

For surgical planning of the fracture, we used the TraumaTech software facilitating virtual reconstruction (both manual and automatic), surgery planning, design of the implant, planning of screw placement and lengths, and production of a 3D print model of the fracture and the implant. The software also supported ordering such custom-made plate from a plate producer who was capable of fast and precise production of the plate.

**Results:**

The surgery using the custom-ordered plate was successful. The actual used screw lengths did not differ from the planned ones by more than 2 mm. One year after the surgery, the patient was capable of more demanding activities and doing sports activities.

**Conclusion:**

This approach provides a great way to prevent complications of the surgery and to shorten its duration. To the best of our knowledge, this is the first description of the treatment of a scapula comminuted fracture utilizing computer-assisted preoperative planning.

## 1 Introduction

Choosing the method of bone fracture treatment is subject to a number of factors. Fractures of long bones in diaphysis heal differently from those in the metaphysis and from fractures of bones without the medullary cavity. The shape and character of the fracture also influence the process of healing. Similarly, healing of simple non-displaced fractures differs from that of multifragmentary and comminuted fractures with significant displacement. Last but not least, the process is also influenced by the general health of the patient, his/her physical condition, and ability to cooperate during the process of bone healing. Precise fracture reduction and stable fragment fixation utilizing new osteosynthesis techniques and modern implant types are at the forefront of the interest of trauma surgeons worldwide.

Since individual bones significantly differ in shape, nowadays implants are tailored to the shape of the site. The choice of the optimal size and shape of the implant should be made prior to the surgery itself and is usually based on the surgeon’s experience, habitual practices of the department, and available scientific information. In the last few years, the use of computer programs capable of modeling the virtual reduction of the fracture is on the rise and such programs are increasingly utilized for planning the optimal shapes and sizes of individual parts of the osteosynthesis implant as well as of their optimal placement, taking into account the biomechanical features of the fractured bone [1,2].

In this article, we present the use of computer assistance for the preoperative planning of a comminuted scapula fracture. To the best of our knowledge, no article describing the use of computer-assisted preoperative planning including 3D print and plate design focusing on scapular fracture has been published so far.

## 2 Case presentation

A 42-year-old patient with a comminuted scapula fracture after falling off a bike was brought to our department and referred for osteosynthesis by plate. The fracture was multifragmentary, it penetrated the neck of the scapula, the lateral edge, and the body of the scapula, and it was significantly dislocated. Therefore, it was necessary to plan a suitable shape and size of the implant for osteosynthesis preoperatively.

**Figure 1:**
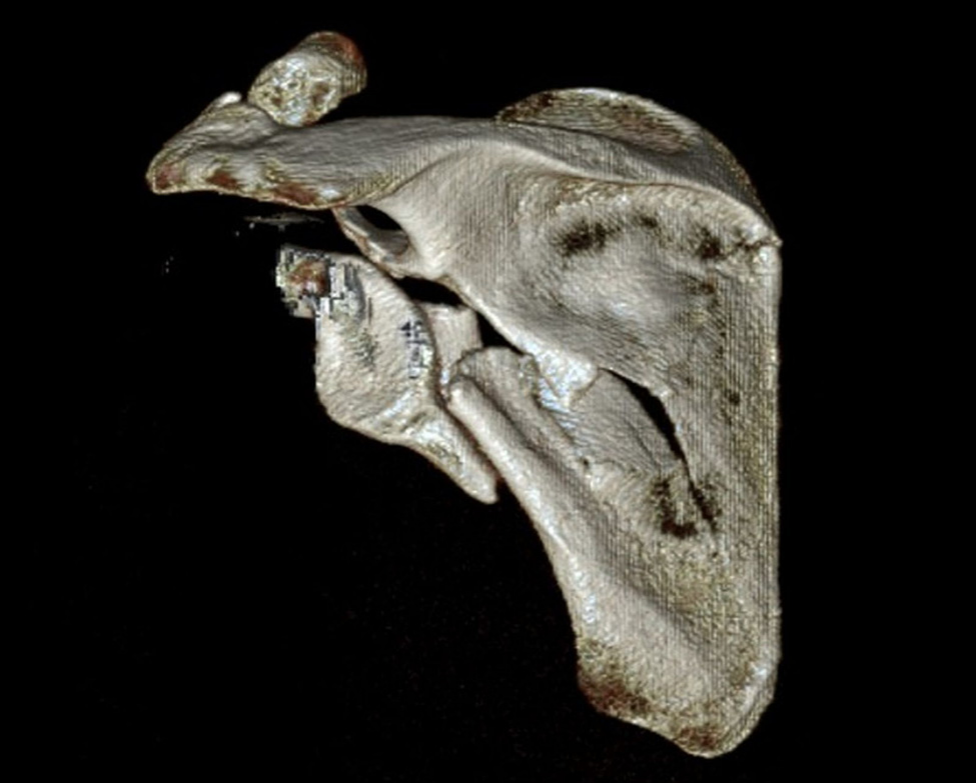
Left scapular fracture – 3D CT reconstruction.

For preoperative planning, we used the TraumaTech software. The program uses data from individual patients’ CT scans (Figure 1) to create a 3D reconstruction of the fracture, including highlighting and color-coding the individual fragments of the fracture, and supports 3D printing of both the fractured bones and the implant.

**Figure 2:**
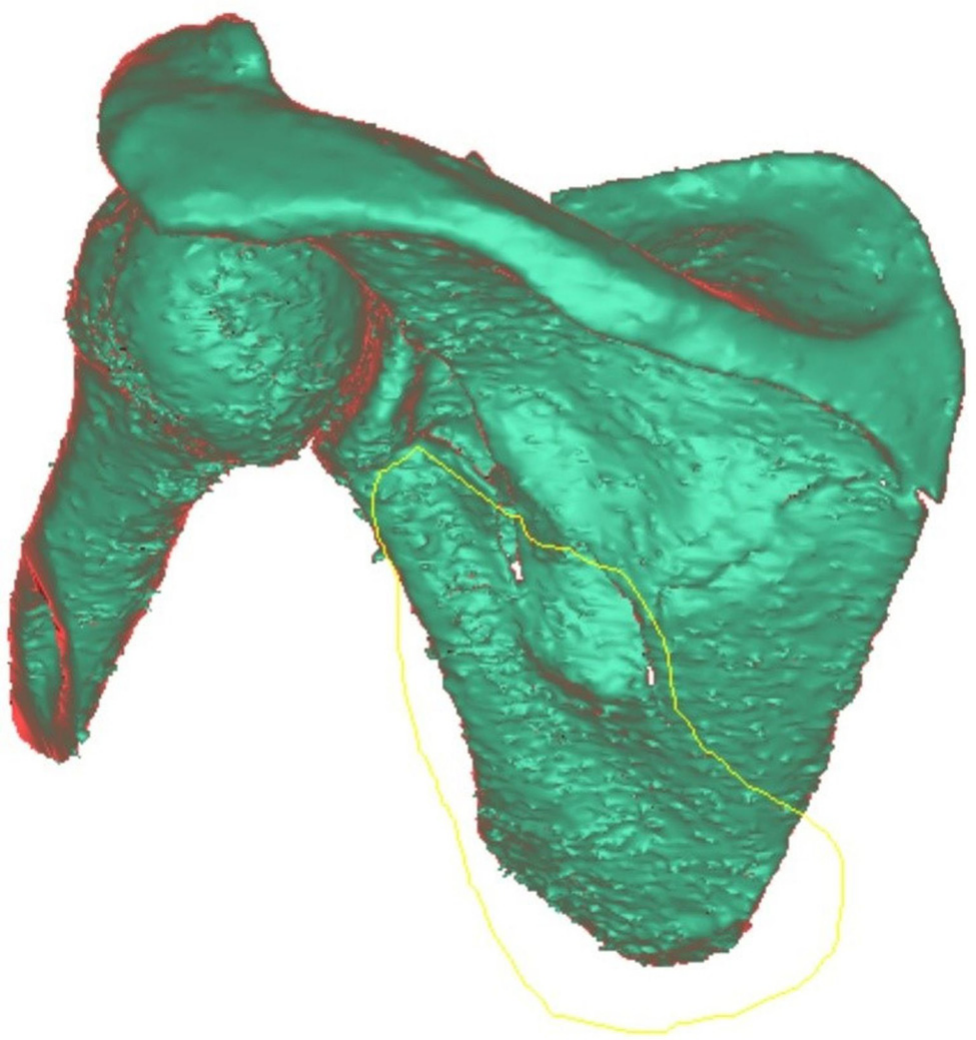
Manual rough delineation of the fracture fragments.

**Figure 3:**
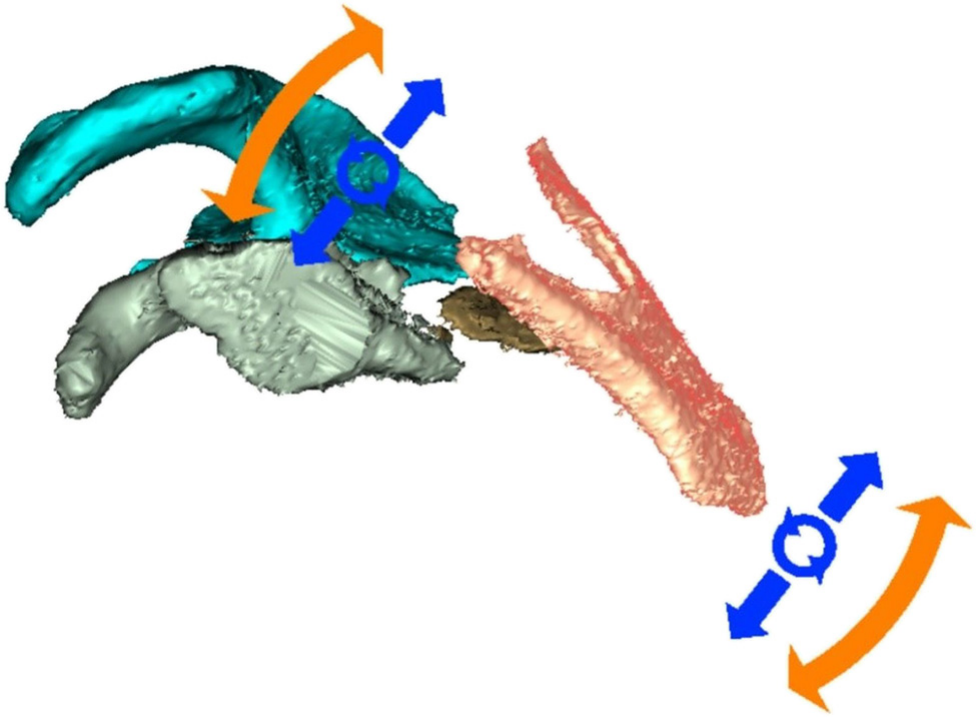
Manual reduction of the fragments.

**Figure 4:**
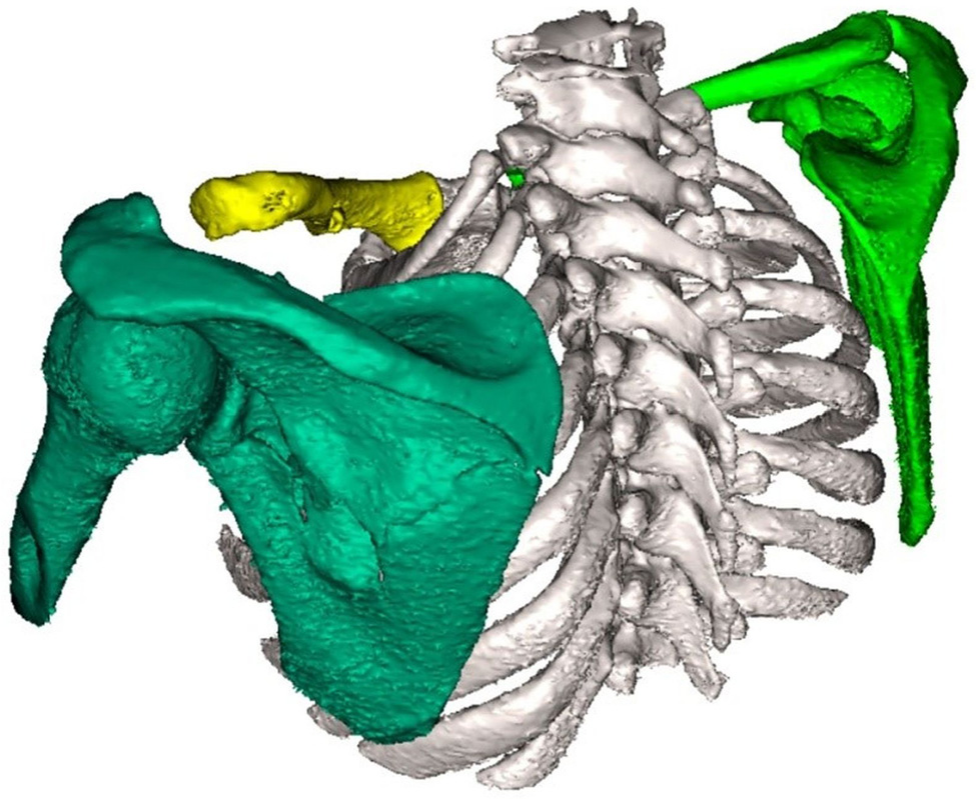
Mirroring the image.

First, individual fragments were manually roughly outlined in the software (relatively accurately where the fragments were present in the bone and only very roughly where there was empty space around the fragment; Figure 2). Subsequently, we assessed two methods of virtual fracture reduction: (I) manual virtual reduction of the fracture to the original anatomical shape or its nearest form (Figure 3) and (II) image mirroring offered by the software (using the paired bone, in this case, the other scapula, as a template for automatic remodeling; Figure 4). Both methods yielded equivalent results with the automated method being significantly faster.

**Figure 5:**
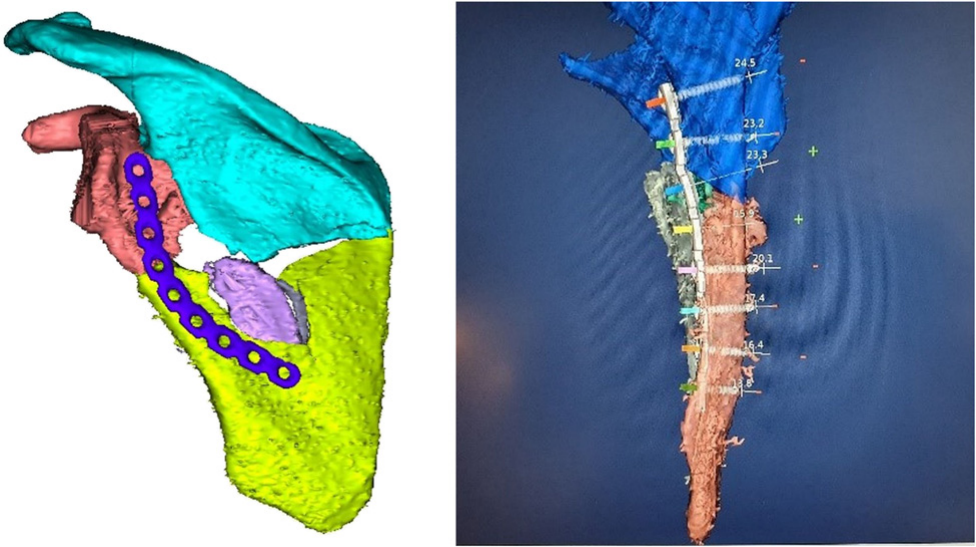
Scapular fracture after the reduction in the TraumaTech software, including optimizing of the implant shape (left) and modeling of the fixation screws (right).

**Figure 6:**
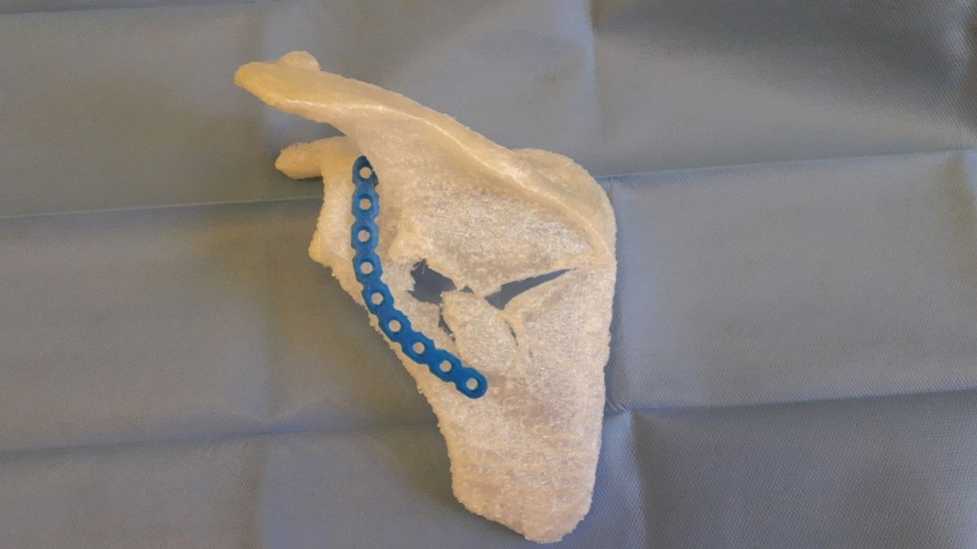
3D print of the scapula model and the implant.

Subsequently, we virtually applied, resized, and reshaped the osteosynthesis implant to optimize it for the planned osteosynthesis (Figure 5a). A virtual plate was pre-formed to the optimal shape and size and applied to the scapula. Subsequently, individual fixation screws and their length were planned in the computer program (Figure 5b). After that, the virtually reduced fractured scapula as well as the plate were 3D printed (Figure 6).

**Figure 7:**
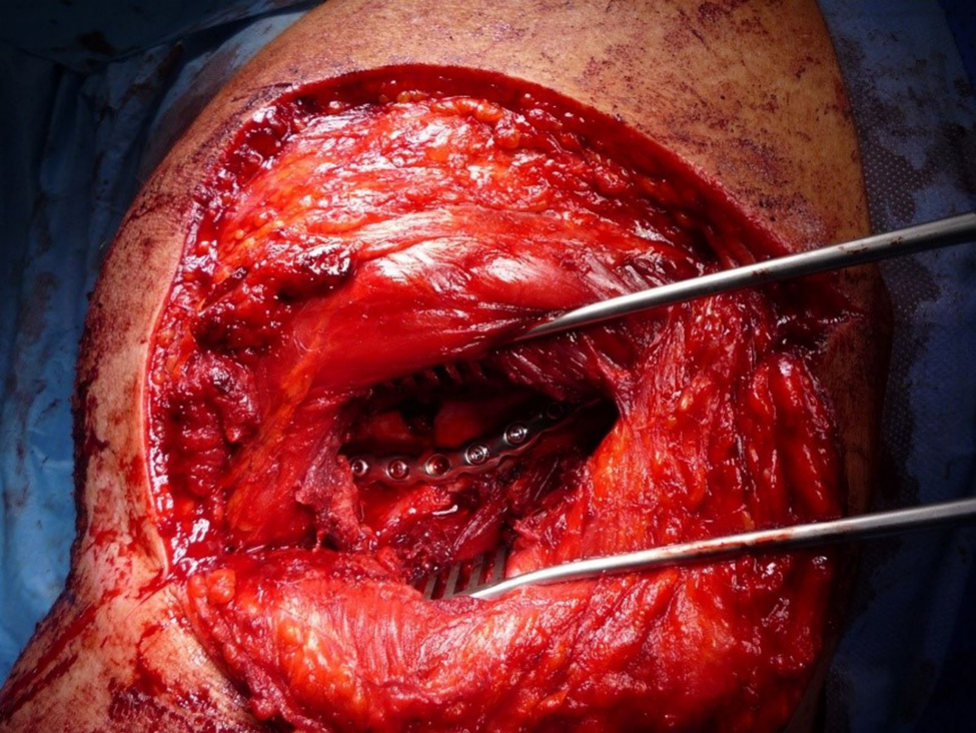
Osteosynthesis of the scapula using a pre-shaped implant.

**Figure 8:**
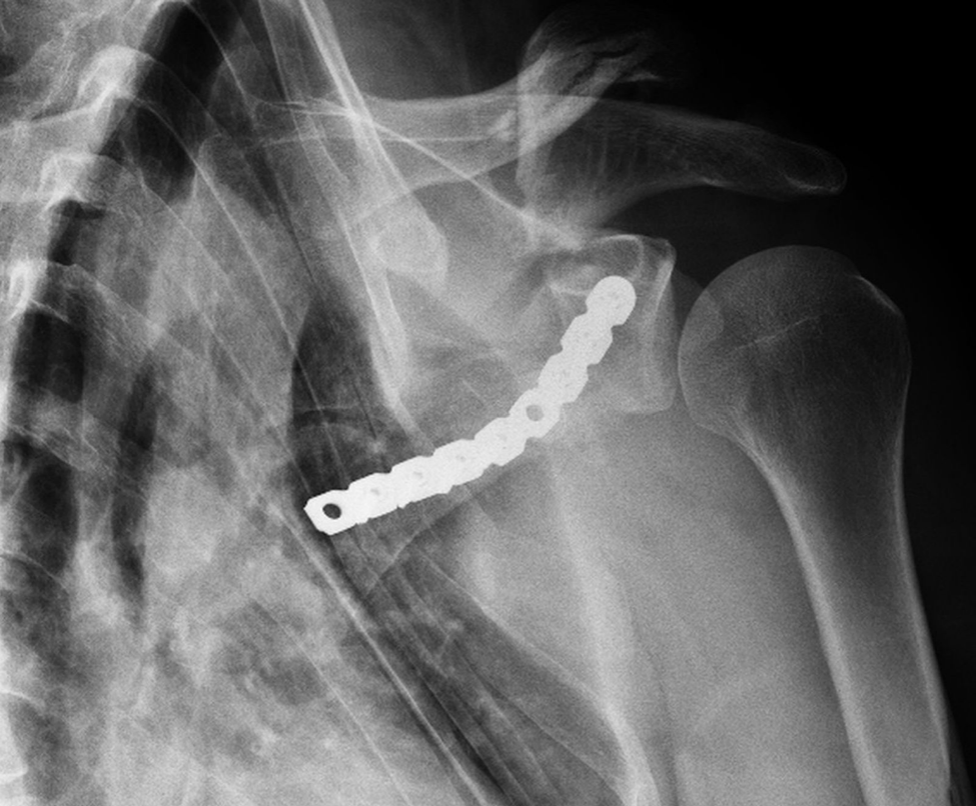
Post-surgery X-ray.

A plate shaped into the pre-planned shape has been custom-made in accordance with the pre-operative planning and printing by a plate manufacturer (Medin a.s., Czech Republic) within two days and used in the osteosynthesis. A dorsal approach to the lateral edge and scapula scoop was used (a modification of the Boyd approach). After the preparation of soft tissue, the scapular fracture was reduced to the pre-planned shape. Subsequently, the plate was placed on the lateral edge, its proximal part penetrating the neck of the scapula and therefore fixating the crucial part of the scapular fracture (Figure 7). The planned screw lengths differed from the actually used ones measured post-operatively by no more than 2 mm. The post-surgery X-ray is shown in Figure 8.

After the surgery, a shoulder orthosis was applied for four weeks, which was followed by rehabilitation. Sixteen weeks after the surgery, the X-ray showed a healed fracture. The clinical condition of the patient stabilized with the range of motion S 40-0-160, F 90-0-0, and R 30-0-80 (ISOM). The patient was capable of normal function and complained only about occasional mild pain (not necessitating the use of analgetics). One year after the surgery, the range of motion was similar, the patient was capable of more demanding activities and doing sports activities. It is over two years since the injury now and the patient’s condition is stable.


**Informed consent:** Informed consent has been obtained from patient included in this study.

## 3 Discussion

Multiple software solutions are currently available for preoperative planning of the osteosynthesis. Most commonly, this technique (including planning shapes of individual implants and their subsequent formation) is used in neurosurgery, maxillofacial surgery, or for filling bone defects in oncological and other indications [3,4,5,6]. In their study on preoperative planning of microvascular mandibular replacement, Gil et al. observed shortening of the surgical time and improvement in the accuracy of the mandibular reconstruction when using computer-assisted preoperative planning [7]. In the field of extremity trauma, preoperational planning is most commonly used in pelvic fractures, but seldom used in long bone fractures and others [8,9,10]. The available software solutions are typically able to carry out a virtual reduction of the fracture, to mark individual fragments, and to optimally place the implant [11,12,13]. The state-of-the-art trend is to use custom-made implants adapted to the shape and anatomical situation of the patient (based on CT scans and 3D virtual reconstruction). The respective anatomical area is then 3D printed and the shaping of the implant is based on the model [14,15], which is a technique we used in this case as well. A cooperation of the surgeons with the plate producer, facilitating the fast production of the plate based on the software-determined parameters is highly beneficial and we believe that this is the path that in the future can improve the care for patients with complicated trauma. In our case, the implant was produced and delivered to our Department within 48 h of ordering, which is an excellent turnaround time. Of course, should the delivery period be too long, we would still be able to utilize the modeling output in producing the implant in-house from prefabricated components. However, such a plate prepared on-site could have inferior mechanical properties (strength) and bending in two planes, which was necessary in our case, is always tricky and may not result in the optimal shape of the plate. In view of these potential problems and the short implant delivery period, we can say that the adopted approach was optimal in the treatment of this particular patient. In the treatment of patients with simpler fractures, the pre-operative in-house production of the implant based on the software model could represent an optimal solution, which would be cheaper and still help in shortening the surgery itself. This aspect is further supported by the fact that proper preoperative planning can take place and the surgeon can prepare a much better surgical plan than on the basis of CT only.

Another aspect that should be noted is getting an idea of the length of the screws that would be needed. The agreement between the predicted and used screws was excellent in our case. This, however, may not be always true as when using other types of plates, the angles of screws can differ from the assumed ones, which can lead to the need for different screws from those predicted. In our case where we used an LCP plate, however, the angles were well defined so this was not an issue.

## 4 Conclusion

The used software for preoperative planning allowed us to create the implant of optimal shape for the osteosynthesis of this complicated scapular fracture. The 3D print of the implant and the scapular fracture after reduction facilitated better preoperative consultation of the operational approach and biomechanical properties of the osteosynthesis. The entire process shortened the time of the surgery itself as the intraoperative shaping of the suitable implant from the standard shape was not necessary.

## References

[1] Birkfellner W, Burgstaller W, Wirth J, Baumann B, Jacob AL, Bieri K, et al. LORENZ: a system for planning long-bone fracture reduction. Medical imaging: visualization, image-guided procedures, and display. Bellingham: SPIE; 2003. p. 500–3.

[2] Jiménez-Delgado JJ, Paulano-Godino F, PulidoRam-Ramírez R, Jiménez-Pérez JR. Computer assisted preoperative planning of bone fracture reduction: simulation techniques and new trends. Med Image Anal. 2016;30:30–45. 10.1016/j.media.2015.12.005 26849422

[3] Guo SS, Zhou WN, Wan LZ, Yuan H, Yuan Y, Du YF, et al. Computer-aided design-based preoperative planning of screw osteosynthesis for type B condylar head fractures: a preliminary study. J Craniomaxillofac Surg. 2016;44:167–76. 10.1016/j.jcms.2015.11.013 26732638

[4] Marmulla R, Niederdellmann H. Computer-assisted bone segment navigation. J Craniomaxillofac Surg. 1998;26:347–59. 10.1016/s1010-5182(98)80067-x 10036650

[5] Mommaerts MY, Jans G, Vander Sloten J, Staels PF, Van der Perre G, Gobin R. On the assets of CAD planning for craniosynostosis surgery. J Craniofac Surg. 2001;12:547–54. 10.1097/00001665-200111000-00008 11711821

[6] Klug C, Schicho K, Ploder O, Yerit K, Watzinger F, Ewers R, et al. Point-to-point computer-assisted navigation for precise transfer of planned zygoma osteotomies from the stereolithographic model into reality. J Oral Maxillofac Surg. 2006;64(3):550–9. 10.1016/j.joms.2005.11.024 16487823

[7] Gil RS, Roig AM, Obispo CA, Morla A, Pagès CM, Perez JL. Surgical planning and microvascular reconstruction of the mandible with a fibular flap using computer-aided design, rapid prototype modelling, and precontoured titanium reconstruction plates: a prospective study. Br J Oral Maxillofac Surg. 2015;53:49–53. 10.1016/j.bjoms.2014.09.015 25305795

[8] Fornaro J, Keel M, Harders M, Marincek B, Székely G, Frauenfelder T. An interactive surgical planning tool for acetabular fractures: initial results. J Orthop Surg Res. 2010;5:50. 10.1186/1749-799X-5-50 PMC2923114 20684761

[9] Boudissa M, Courvoisier A, Chabanas M, Tonetti J. Computer assisted surgery in preoperative planning of acetabular fracture surgery: state of the art. Expert Rev Med Devices. 2018;15:81–9. 10.1080/17434440.2017.1413347 29206497

[10] Chen Y, Zhang K, Qiang M, Li H, Dai H. Computer-assisted preoperative planning for proximal humeral fractures by minimally invasive plate osteosynthesis. Chin Med J. 2014;127(18):3278–85. 25266527

[11] Kamer L, Nötzli C, Erdöhelyi B. Method for generating a graphical 3d computer model of at least one anatomical structure in a selectable pre-, intra-, or postoperative status; 2015. U.S. Patent Application No 14/414367.

[12] Kovler I, Joskowicz L, Weil YA, Khoury A, Kronman A, Mosheiff R, et al. Haptic computer-assisted patient-specific preoperative planning for orthopedic fractures surgery. Int J Comput Assist Radiol Surg. 2015;10:1535–46. 10.1007/s11548-015-1162-9 25749801

[13] Verhamme LM, Meijer GJ, Soehardi A, Bergé SJ, Xi T, Maal TJJ. An accuracy study of computer-planned implant placement in the augmented maxilla using osteosynthesis screws. Int J Oral Maxillofac Surg. 2017;46:511–7. 10.1016/j.ijom.2016.10.013 27887876

[14] Wu XB, Wang JQ, Zhao CP, Sun X, Shi Y, Zhang ZA, et al. Printed three-dimensional anatomic templates for virtual preoperative planning before reconstruction of old pelvic injuries: initial results. Chin Med J. 2015;128:477–82. 10.4103/0366-6999.151088 PMC4836250 25673449

[15] Zeng C, Xing W, Wu Z, Huang H, Huang W. A combination of three-dimensional printing and computer-assisted virtual surgical procedure for preoperative planning of acetabular fracture reduction. Injury. 2016;47:2223–7. 10.1016/j.injury.2016.03.015 27372187

